# *Aconiti lateralis Radix Praeparata* inhibits Alzheimer’s disease by regulating the complex regulation network with the core of *GRIN1* and *MAPK1*

**DOI:** 10.1080/13880209.2021.1900879

**Published:** 2021-03-30

**Authors:** Yutao Wang, Huixiang Zhang, Jing Wang, Ming Yu, Qianqian Zhang, Shan Yan, Dingyun You, Lanlan Shi, Lihuan Zhang, Limei Wang, Hongxiang Wu, Xue Cao

**Affiliations:** a Department of Laboratory Animal Science, Kunming Medical University, Kunming, China; b Basic Medical College, Kunming Medical University, Kunming, China; c Institute of Neuroscience, Basic Medical College, Kunming Medical University, Kunming, China; d College of Veterinary Medicine, Yunnan Agricultural University, Kunming, China; e Yunnan Key Laboratory of Stem Cell and Regenerative Medicine, Bioengineering Centre, Kunming Medical University, Kunming, P.R. China; f School of Public Health, Kunming Medical University, Kunming, China; g Faculty of Rehabilitation Medicine, Kunming Medical University, Kunming, China

**Keywords:** Fuzi, therapy, APP cells, aminophenol

## Abstract

**Context:**

Current medicine for Alzheimer’s disease (AD) cannot effectively reverse or block nerve injury. Traditional Chinese Medicine practice and research imply *Aconiti lateralis Radix Praeparata* (Fuzi) may meet this goal.

**Objective:**

Analysing the anti-AD effect of Fuzi and its potential molecular mechanism.

**Materials and methods:**

AD model cells were treated with Fuzi in 0-300 mg/mL for 24 h in 37 °C. The cell viability (CV) and length of cell projections (LCP) for each group were observed, analysed, and standardised using control as a baseline (CV_s_ and LCP_s_). The Fuzi and AD relevant genes were identified basing on databases, and the molecular mechanism of Fuzi anti-AD was predicted by network analysis.

**Results:**

Experiment results showed that Fuzi in 0.4 mg/mL boosted LCP (LCP_s_ = 1.2533, *p* ≤ 0.05), and in 1.6–100 mg/mL increased CV (CV_s_ from 1.1673 to 1.3321, *p* ≤ 0.05). Bioinformatics analysis found 17 Fuzi target genes (relevant scores ≥ 20), showing strong AD relevant signals (RMS_*p* ≤ 0.05, related scores ≥ 5), enriched in the pathways regulating axon growth, synaptic plasticity, cell survival, proliferation, apoptosis, and death (*p* ≤ 0.05). Especially, *GRIN1* and *MAPK1* interacted with *APP* protein and located in the key point of the “Alzheimer’s disease” pathway.

**Discussion and conclusions:**

These results suggest that Fuzi may have therapeutic and prevention potential in AD, and *GRIN1* and *MAPK1* may be the core of the pathways of the Fuzi anti-AD process. Fuzi should be studied more extensively, especially for the prevention of AD.

## Introduction

Alzheimer’s disease (AD), an age-related, neurodegenerative disease, causes progressive memory decline and cognitive dysfunction (Lane et al. 2018). The incidence rate of this disease is increasing each year, and it is estimated that more than 80 million patients worldwide will suffer from this disease by 2050 (Mangialasche et al. 2010; Prince et al. 2013; Van Cauwenberghe et al. 2016). However, the treatment of AD still plagues us, due to its complex pathogenesis and limited types of available drugs (Mangialasche et al. 2010; Briggs et al. 2016; De Strooper and Karran 2016). More seriously, these drugs can’t reverse or block the nervous system injury (Briggs et al. 2016; Lee and Kim 2017), and there is lack of high-quality evidence for their efficacy in mild cognitive impairment (Anderson 2019). So, other drugs are needed to reverse or block the nervous system damaged in AD, even in the mild cognitive impairment (MCI) stage.

Although the mechanism is not clear, Traditional Chinese Medicines (TCM) have been applied to AD treatment in practice in China to address both the symptoms and root causes with good effectiveness, one of which is *Aconiti lateralis Radix Praeparata* (Fuzi). Previous studies have confirmed that Fuzi-based prescriptions combining with existing AD drugs improve cognitive status effectively in AD patients (Yang et al. 2018) and inhibit nerve cell apoptosis (Sheng et al. 2004; Sun et al. 2018). Many ingredients of Fuzi, such as *EIC,* myristic acid, deltoin, etc., target to muscarinic acetylcholine receptor M1, cholinesterase and acetylcholinesterase (Ru et al. 2014), which play important roles in AD relevant processes: learning and memory processes, the metabolism of amyloid-β precursor (*APP*) protein, and neurotransmission (Ahmed et al. 2017; Lazarevic-Pasti et al. 2017; Felder et al. 2018; Scarpa et al. 2020). Moreover, asiatic acid, another component of Fuzi, plays an favorable role in the protection of brain nerves (Sirichoat et al. 2015; Chaisawang et al. 2017; Park et al. 2017; Ahmad Rather et al. 2018; Loganathan and Thayumanavan 2018). Fuzi protects the mitochondria (Kang et al. 2016; Lu et al. 2017; Huang et al. 2018) whose injury plays an important role in AD development. Fuzi has anti-aging effects by enhancing the antioxidant capacity and inhibiting cell apoptosis (Zhou et al. 2015), which is a prominent risk factor for AD (Trevisan et al. 2019).

The above information suggests that Fuzi, a multi-target Chinese herb, could be used as an anti-AD medicine. It may treat AD by anti-aging, and protecting mitochondria and nervous system. To study how Fuzi performs in anti-AD, we performed cellular experiments and bioinformatics analysis in this research. The results show that Fuzi is a promising medicine for AD treatment and protection.

## Materials and methods

### Culture of APP cells

SH-SY5Y cell line, over-expressing 695-amino-acid Swedish mutation amyloid-β precursor protein (*APP* cell) (Kong et al. 2015; Song et al. 2015), was kindly provided from Wang’s lab (Department of Laboratory Animal Science, Kunming Medical University, Kunming, China), which had been established and its effectiveness had been tested in 2017. The cells were cultured in DMEM medium (Hyclone, code: DMEIF-12 (AF29527017)) with 10% foetal bovine serum (Gibco, code: 2139378 cp), in 37 °C and 5% CO_2_ condition. The cells were resuscitated and identified before the experiment. The APP cells passing the check for contamination were used in the follow-up experiment.

### CCK-8 method to detect cell viability

Fuzi granules extracts (Guangdong Pharmaceutical Co., LTD.) were dissolved in DMEM medium (Hyclone, code: DMEIF-12 (AF29527017)) with 1% foetal bovine serum (Gibco, code: 2139378 cp) to a final concentration of 400 mg/mL. APP cell was seeded in a 96-well plate with 5000 cells/well. The drug was administrated when cells grew to 70% confluence. The culture contained Fuzi with final concentration of 0, 0.4, 0.8, 1.6, 3.125, 6.25, 12.5, 25, 50, 100, 200, 250, and 300 mg/mL was used to replace the basal culture medium and the cell was cultured for 24 h. CCK-8 solution (10 μL) (Cell Counting Kit-8 of Wanleibo, code: WLP004) was added to each well and incubated for 1–4 h, then. The absorbance at 450 nm was determined by a microplate reader (BIOTEK, code: ELX-800). The cell viability that represents cell proliferation activity was calculated as below:

$$\text{Cell viability }(\%)=[A_{\mathrm{dose}}-A_{\mathrm{blank}}]/[A_{0\;\mathrm{dose}}-A_{\mathrm{blank}}]\times 100\%$$

*A*_dose_: Absorbance of cells, CCK solution, and drug solution *A*_blank_: Absorbance of the medium, CCK solution, but no cells *A*_0 dose_: Absorbance of cells, CCK solution, but no drug solution.

The cell viability (CV) of the cells treated without Fuzi (NC) was used as a baseline to standardise that of other groups, which were symbolised as CV (Supplementary Material).

### Observation of cell morphology

When the APP cells were grown to about 70% confluence, culture containing 0, 0.4, 0.8, 1.6, 3.125, 6.25, 12.5, 25, 50, 100, 200, 250, 300 mg/mL of Fuzi replaced the basal culture medium. The cells were observed under inverted phase contrast microscope (Nikon, code: TE2000-U) after cultured for 24 h in 37 °C, 5% CO_2_ condition.

### Measurement of the length of projections (LCP)

The total number and LCP in a visual field were measured by Viewpoint (BETA v1.0.0.0) software. The average projections length in the visual field was obtained by dividing the total LCP with the total cell number. Three visual fields were measured in each group, which were averaged to obtain the final LCP. LCP of different groups were standardised using NC as a baseline (Supplementary Material), which were symbolised as LCP_s_. The summary graphs were drawn with ImageJ and Adobe Illustrator.

### Statistical analyzes

The data of CV_s_ and LCP_s_ were analysed by SPSS17.0. Multiple group comparisons were performed using one-way analysis of variance (ANOVA). After homogeneity of variances test, Fisher’s least significant difference (LSD) or Dunnett’s T3 test (uneven) were performed to determine significant differences between different groups. Data was expressed as means ± SD, and *p* ≤ 0.05 (*) and *p* ≤ 0.01 (**) was considered as statistically and extremely significant. The statistical analysis of enrichment analysis used the Fisher's test of the database.

### Bioinformatics analyses

The related ingredients, diseases, and target genes were informed with keyword Fuzi in the Traditional Chinese Medicine systems pharmacology (TCMSP) database (http://tcmspw.com/tcmsp.php) (Ru et al. 2014) and summarised with the AD-related information. The ingredients of Fuzi and their target genes were searched with key words of Fuzi and Fupian (synonym for Fuzi) and filtered using score ≥ 10 and adjusted *p*-value ≤ 0.05 (*p*-value after Benjamini-Hochberg multiple testing correction) as the cut-off in the BATMAN-TCM database (http://bionet.ncpsb.org/batman-tcm/) (Liu et al. 2016). Then the genes with scores higher than five were selected to calculate root mean square (RMS) (Wang et al. 2014) of multiple correlation values for the same ingredients and same genes, in which genes with RMS score higher than 20 were defined as target genes of Fuzi. The ‘Alzheimer’s disease’ was searched as the keyword in the GeneCards database (http://www.genecards.org/) (Stelzer et al. 2016), and the relevant genes with a score higher than 10 were selected as AD correlated genes.

The UK Biobank and IGAP meta-analysis GWAS summary statistic of AD was download from the Psychiatric Genomics Consortium (http://web.pasteur-lille.fr/en/recherche/u744/igap/igap_download.php) (Lambert et al. 2013) and the SNPs with significant *p* values (*p* ≤ 0.05) were annotated by Annovar. The GEO data (GSE1297, GSE36980, GSE44772, GSE48350 and GSE5281) (Blalock et al. 2004; Liang et al. 2007; Berchtold et al. 2008; Zhang et al. 2013; Hokama et al. 2014) of Human AD expression profile was analysed by GEO2R for differential expression (Naoi et al. 2004), where different tissues were analysed independently. RMS of *p* values was calculated after genes were screened with the condition of *p* ≤ 0.05 in each independent analysis; candidate genes were selected with the condition of RMS_*p* ≤ 0.05 and the condition of *p* ≤ 0.05 in five independent analysis. Finally, the overlapping genes of the above analysis were obtained by the Perl script. String (https://string-db.org/) (Szklarczyk et al. 2019) and Reactome (http://reactome.org/) (Fabregat et al. 2018) were performed for protein interaction and pathway enrichment analysis, respectively. The summary graphs were drawn with R version 3.4.3 and Adobe Illustrator.

All candidate genes were located in the classical AD pathway by tools of the KEGG database (http://www.kegg.jp/) (Ghansah et al. 1993). The candidate genes relevant ingredients of Fuzi were figured out from the predicted list download from the BATMAN-TCM database (Liu et al. 2016) by Perl script. The Venn diagram was also drawn with R version 3.4.3 and Adobe Illustrator.

## Results

### The impact of Fuzi on cell changes with its different concentration

**Figure 1.**
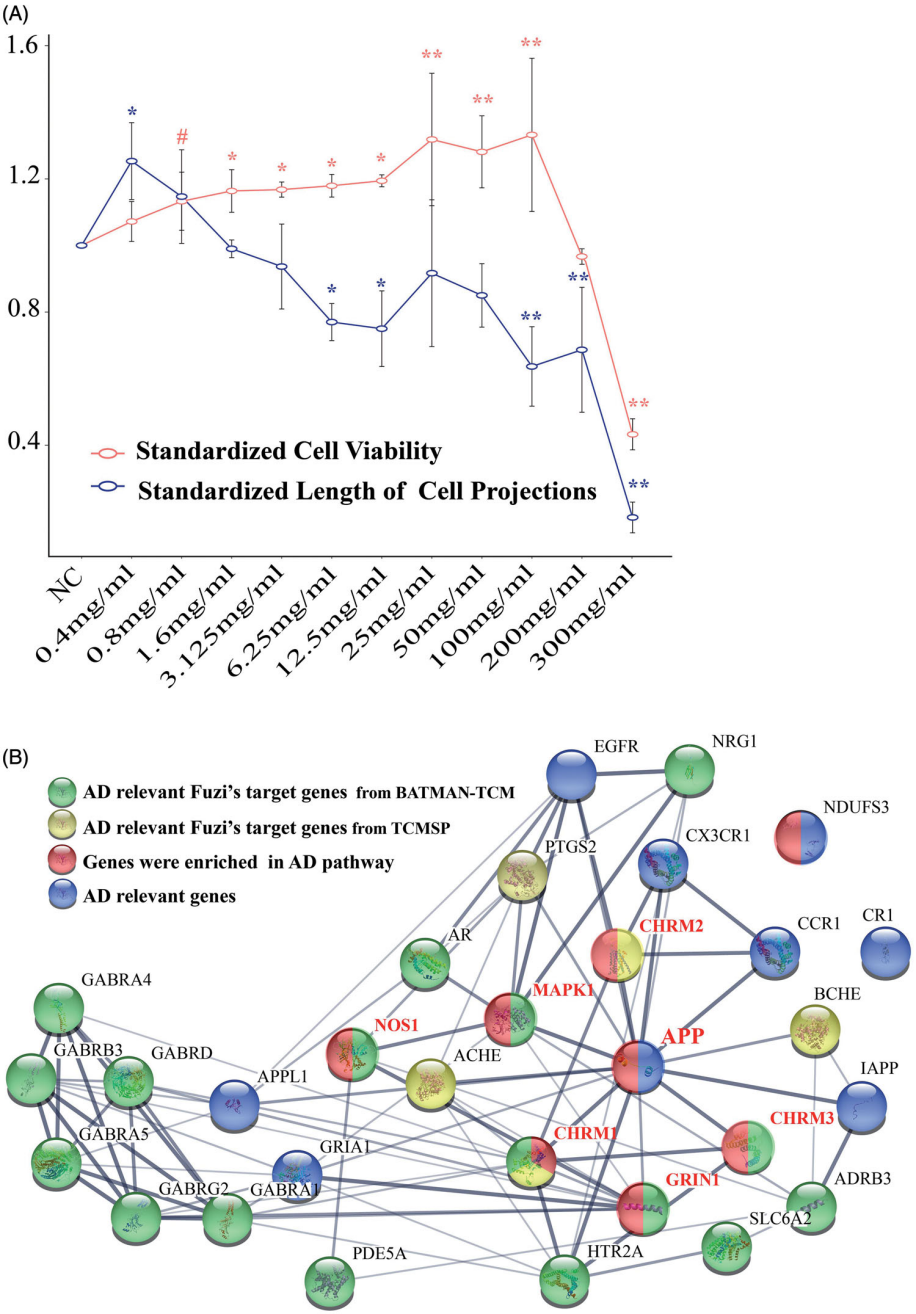
The gene interaction and the change pattern of cells. (A) The modification pattern for the cell viability and the length of cell’s projections, when APP cells were treated by Fuzi solution of different concentration. Statistically (*p* ≤ 0.05) and extremely (*p* ≤ 0.01) significant difference were symbolled as “*” and “**”, and *p* ≤ 0.1 was symbolled as “#”. (B) The gene interaction pattern for the AD relevant Fuzi target genes.

APP cell line as a classical cell model of AD (Kong et al. 2015; Song et al. 2015), were treated with the solution of Fuzi granules at a gradient concentration (0.4–300 mg/mL) for 24 h, then CV and LCP were analysed. The results showed that APP cells treated with a suitable concentration of Fuzi not only increased CV but also promoted LCP. However, when the cells were treated with an over-high concentration solution of Fuzi granules treatment, both the CV and LCP decreased significantly (Figure 1(A)).

The CV of APP cells increased slowly when Fuzi concentration augmented from 0.4–12.5 mg/mL (CV_s_ from 1.0719 to 1.1942), and from 1.6 mg/mL (CV_s_ = 1.1673) the differences in the CV of APP cells treated with and without Fuzi (NC) reached a significant level (*p* ≤ 0.05). CV increased sharply in 25 mg/mL (CV_s_ = 1.3182) and reached a peak at 100 mg/mL (CV_s_ = 1.3321). In these concentrations, the CV of APP cells was extremely higher than that in NC (*p* ≤ 0.01). It plunged, then, when the concentration continued to increase, which decreased significantly comparing to NC when concentration was in more than 200 mg/mL (CV_s_ = 0.9667, *p* ≤ 0.01, Figure 1(A)).

**Figure 2.**
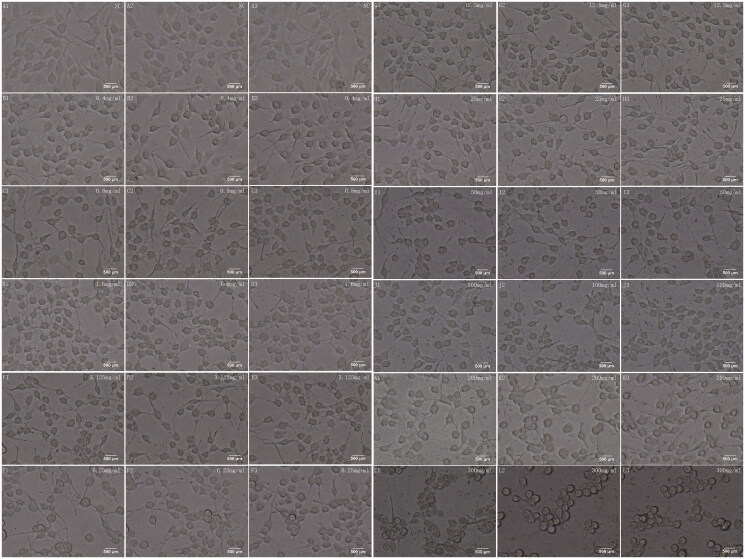
The microscope images for cell morphology. NC symbol the APP cells were not treated by Fuzi. The other images symbol the APP cells were treated by Fuzi solution of different concentration, from 0.4 to 300 mg/ml.

Comparatively, LCP were promoted in 0.4 mg/mL (LCP_s_ = 1.2533) and 0.8 mg/mL (LCP_s_ = 1.1467) Fuzi treated groups (Figure 1(A)), and the cells were in healthy spindle shape (Figure 2(B–C)). Especially in the 0.4 mg/mL group, LCP of APP cells was significantly higher than that of the NC group (*p* ≤ 0.05, Figure 1(A)). Notably, it even show the opposite effect when the concentration was higher than 0.8 mg/mL. Although LCP increased a little at some Fuzi concentration, the processes of APP cells treated with Fuzi tended to be shorter than that of NC starting from 1.6 mg/mL (LCP_s_ = 0.9900, Figure 1(A)). The APP cells’ processes became extremely short when the concentration was more than 25 mg/mL (LCP_s_ = 0.9167, *p* < 0.01, Figure 1(A)), and the cell shape also turned from a spindle shape to nearly circle or triangle (Figure 2(H-L)).

### The molecular mechanism of Fuzi’s anti-AD function is revealed in bioinformatics analysis

**Figure 3.**
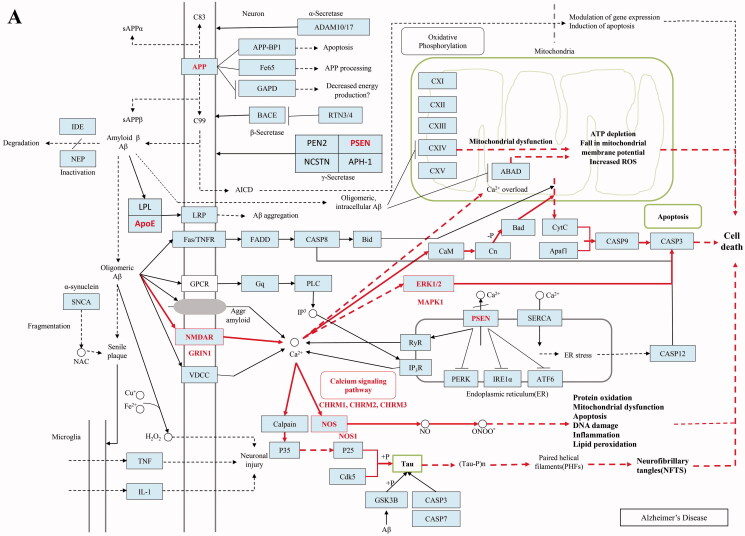
The KEGG pathway of AD.

To reveal the molecular mechanism of Fuzi’s anti-AD function, the target genes of Fuzi were predicted by the tools of BATMAN-TCM database (Liu et al. 2016), and the AD relevant genes were obtained based on the analysis for the data from Psychiatric Genomics Consortium (Lambert et al. 2013), GEO (Naoi et al. 2004), and GeneCards (Stelzer et al. 2016) databases. As showed in materials and methods, we performed the analysis and found 17 target genes of Fuzi (relevant scores ≥ 20). The 17 target genes significantly differentially expressed between AD and normal samples (RMS_*p* ≤ 0.05), own high related scores (related scores ≥ 5) for AD in the GeneCards database (Stelzer et al. 2016) (Table 1), and 15 of them contained significant SNP sites in AD GWAS analysis. Moreover, the 17 genes are involved in the amyloid-β precursor protein (APP) interaction network with other AD relevant genes (Figure 1(B)), which were defined as methods showing. Importantly, five target genes of Fuzi (Cholinergic Receptor Muscarinic 1/3: *CHRM1/3*, Glutamate Ionotropic Receptor NMDA Type Subunit 1: *GRIN1*, Mitogen-Activated Protein Kinase 1: *MAPK1*, Nitric Oxide Synthase 1: *NOS1*) were enriched in the classical AD pathway of KEGG (Figure 1(B), Table 1). They were located in the important position of the pathway (Figure 3), which are responsible for the regulation of mitochondrial dysfunction, apoptosis, neurofibrillary tangles, cell death, protein oxidation, DNA damage, inflammation and lipid peroxidation (Figure 3).

**Table 1.** The information for the 17 AD relevant target genes of Fuzi.

Gene Symbol	Gene Name	Related Scores for AD^1^	RMS_P values of DEG^2^	The Highest Related Scores for the Fuzi’s Ingredients^3^
ADRB3	Adrenoceptor Beta 3	5.39	0.020355572	122.778
AR	Androgen Receptor	9.49	0.034383704	48
**CHRM1**	**Cholinergic Receptor Muscarinic 1**	**7.82**	**0.021075246**	**48**
**CHRM3**	**Cholinergic Receptor Muscarinic 3**	**13.88**	**0.028798586**	**48**
GABRA1	Gamma-Aminobutyric Acid Type A Receptor Alpha1 Subunit	17.46	0.020776697	80.882
GABRA4	Gamma-Aminobutyric Acid Type A Receptor Alpha4 Subunit	8.26	0.009644649	80.882
GABRA5	Gamma-Aminobutyric Acid Type A Receptor Alpha5 Subunit	7.93	0.02979358	80.882
GABRB3	Gamma-Aminobutyric Acid Type A Receptor Beta3 Subunit	16.56	0.021044654	80.882
GABRD	Gamma-Aminobutyric Acid Type A Receptor Delta Subunit	13.35	0.018002658	80.882
GABRG2	Gamma-Aminobutyric Acid Type A Receptor Gamma2 Subunit	9.96	0.016433434	80.882
**GRIN1**	**Glutamate Ionotropic Receptor NMDA Type Subunit 1**	**17.06**	**0.022782919**	**22.373**
HTR2A	5-Hydroxytryptamine Receptor 2A	24.18	0.027636956	80.882
**MAPK1**	**Mitogen-Activated Protein Kinase 1**	**11.21**	**0.022727156**	**48**
**NOS1**	**Nitric Oxide Synthase 1**	**9.64**	**0.025594657**	**22.373**
NRG1	Neuregulin 1	5.38	0.018124479	22.373
PDE5A	Phosphodiesterase 5A	5.56	0.020541902	22.373
SLC6A2	Solute Carrier Family 6 Member 2	11.15	0.017844332	48

^1^The related scores for AD showed in GeneCards database. ^2^The RMS_P values were calculated basing on the gene expression data for AD showed in GEO database. ^3^The related scores for the Fuzi’s ingredients were provided by BATMAN-TCM database. The genes enriched in the classical AD pathway of KEEG were highlighted by boldface.

The functional annotation and enrichment analysis were performed by Reactome (Fabregat et al. 2018) for the 17 AD relevant Fuzi’s target genes. Then, the target genes were enriched in the pathway responsible for axon growth (Axon guidance (FDR = 4.70E-02), *L1CAM* interactions (FDR = 1.54E-02), cell apoptotic (Apoptotic factor-mediated response (FDR = 2.51E-02) and Cytochrome c-mediated apoptotic response (FDR = 2.10E-02)), the bi-directional regulation for cell death and proliferation (*MAPK1/MAPK3* signalling (FDR = 8.15E-04)), cell survival (*PIP3* activates *AKT* signalling (FDR = 1.54E-02), signalling by *ERBB2* (FDR = 8.50E-03), signalling by *NTRKs* (FDR = 1.54E-02)) and the function of neuronal system (Neuronal System (FDR = 1.06E-05), transmission across Chemical Synapses (FDR = 1.87E-06), Negative regulation of NMDA receptor-mediated neuronal transmission (FDR = 3.74E-02) and *MECP2* regulates neuronal receptors and channels (FDR = 4.42E-02) (Table 2). Interestingly, the screened genes *MAPK1* and *GRIN1* not only participate the pathways responsible for the function of neurons (Axon guidance, Neurotransmitter receptors, and postsynaptic signal transmission, and so on), but also work in the neuron survival relevant pathways (*MAPK1/MAPK3* signalling, signalling by *ERBB2*, and so on). Moreover, the two participate in half of the pathways.

**Table 2.** The pathway enrichment results of 17 Fuzi’s target genes.

Pathway identifier	Pathway name	Candidate	Total	Ratio	P Value	FDR
**R-HSA-112314**	**Neurotransmitter receptors and postsynaptic signal transmission**	**8**	**232**	**1.60E-02**	**2.75E-10**	**7.71E-08**
**R-HSA-112315**	**Transmission across Chemical Synapses**	**8**	**352**	**2.43E-02**	**7.12E-09**	**9.97E-07**
R-HSA-977443	GABA receptor activation	5	67	4.62E-03	2.31E-08	2.15E-06
R-HSA-1236394	Signalling by ERBB4	5	82	5.65E-03	6.27E-08	4.39E-06
**R-HSA-112316**	**Neuronal System**	**8**	**498**	**3.43E-02**	**1.03E-07**	**5.79E-06**
R-HSA-375280	Amine ligand-binding receptors	4	88	6.06E-03	4.87E-06	2.24E-04
R-HSA-1306955	GRB7 events in ERBB2 signalling	2	6	4.13E-04	2.91E-05	1.02E-03
R-HSA-390648	Muscarinic acetylcholine receptors	2	6	4.13E-04	2.91E-05	1.02E-03
**R-HSA-9006934**	**Signalling by Receptor Tyrosine Kinases**	**6**	**622**	**4.29E-02**	**1.04E-04**	**3.21E-03**
R-HSA-1358803	Downregulation of ERBB2:ERBB3 signalling	2	16	1.10E-03	2.05E-04	5.19E-03
**R-HSA-162582**	**Signal Transduction**	**12**	**3364**	**2.32E-01**	**2.27E-04**	**5.19E-03**
**R-HSA-438064**	**Post NMDA receptor activation events**	**3**	**96**	**6.61E-03**	**2.59E-04**	**5.19E-03**
R-HSA-8847993	ERBB2 Activates PTK6 Signalling	2	18	1.24E-03	2.59E-04	5.19E-03
R-HSA-6785631	ERBB2 Regulates Cell Motility	2	19	1.31E-03	2.89E-04	5.20E-03
**R-HSA-1963642**	**PI3K events in ERBB2 signalling**	**2**	**22**	**1.52E-03**	**3.86E-04**	**6.57E-03**
R-HSA-442755	Activation of NMDA receptors and postsynaptic events	3	114	7.85E-03	4.27E-04	6.65E-03
**R-HSA-416476**	**G alpha (q) signalling events**	**4**	**283**	**1.95E-02**	**4.43E-04**	**6.65E-03**
**R-HSA-5673001**	**RAF/MAP kinase cascade**	**4**	**290**	**2.00E-02**	**4.86E-04**	**6.80E-03**
**R-HSA-5684996**	**MAPK1/MAPK3 signalling**	**4**	**297**	**2.05E-02**	**5.31E-04**	**6.86E-03**
**R-HSA-6811558**	**PI5P, PP2A and IER3 Regulate PI3K/AKT Signalling**	**3**	**126**	**8.68E-03**	**5.71E-04**	**6.86E-03**
**R-HSA-9607240**	**FLT3 Signalling**	**4**	**311**	**2.14E-02**	**6.31E-04**	**7.51E-03**
**R-HSA-199418**	**Negative regulation of the PI3K/AKT network**	**3**	**134**	**9.23E-03**	**6.83E-04**	**7.51E-03**
**R-HSA-9620244**	**Long-term potentiation**	**2**	**31**	**2.14E-03**	**7.61E-04**	**8.38E-03**
**R-HSA-8940973**	**RUNX2 regulates osteoblast differentiation**	**2**	**34**	**2.34E-03**	**9.14E-04**	**9.14E-03**
**R-HSA-5683057**	**MAPK family signalling cascades**	**4**	**348**	**2.40E-02**	**9.60E-04**	**9.21E-03**
R-HSA-9665686	Signalling by ERBB2 TMD/JMD mutants	2	35	2.41E-03	9.68E-04	9.21E-03
R-HSA-1250196	SHC1 events in ERBB2 signalling	2	36	2.48E-03	1.02E-03	9.21E-03
R-HSA-8863795	Downregulation of ERBB2 signalling	2	36	2.48E-03	1.02E-03	9.21E-03
**R-HSA-442742**	**CREB1 phosphorylation through NMDA receptor-mediated activation of RAS signalling**	**2**	**39**	**2.69E-03**	**1.20E-03**	**1.06E-02**
R-HSA-392154	Nitric oxide stimulates guanylate cyclase	2	41	2.82E-03	1.32E-03	1.06E-02
**R-HSA-8941326**	**RUNX2 regulates bone development**	**2**	**43**	**2.96E-03**	**1.45E-03**	**1.16E-02**
R-HSA-9664565	Signalling by ERBB2 KD Mutants	2	46	3.17E-03	1.66E-03	1.33E-02
R-HSA-1227990	Signalling by ERBB2 in Cancer	2	48	3.31E-03	1.80E-03	1.44E-02
R-HSA-373076	Class A/1 (Rhodopsin-like receptors)	4	462	3.18E-02	2.71E-03	1.90E-02
R-HSA-8848021	Signalling by PTK6	2	71	4.89E-03	3.87E-03	2.71E-02
R-HSA-9006927	Signalling by Non-Receptor Tyrosine Kinases	2	71	4.89E-03	3.87E-03	2.71E-02
R-HSA-5619109	Defective SLC6A2 causes orthostatic intolerance (OI)	1	3	2.07E-04	3.92E-03	2.74E-02
R-HSA-1227986	Signalling by ERBB2	2	78	5.37E-03	4.65E-03	3.25E-02
**R-HSA-1257604**	**PIP3 activates AKT signalling**	**3**	**316**	**2.18E-02**	**7.70E-03**	**4.62E-02**
R-HSA-2219530	Constitutive Signalling by Aberrant PI3K in Cancer	2	103	7.10E-03	7.95E-03	4.77E-02

The lines in boldface were the pathways MAPK1 or GRIN1 genes taking part in.

At the same time, the AD relevant target genes of Fuzi were also scanned in the TCMSP database (Ru et al. 2014). Five target genes (Prostaglandin G/H synthase 2: *PTGS2*, Cholinergic Receptor Muscarinic 1/2: *CHRM1/2*, Cholinesterase: *BCHE*, and Acetylcholinesterase: *ACHE*) (Table 3) were found. These genes are also involved in the protein interaction network of AD relevant genes (Figure 1(B)). *CHRM2* and *CHRM1* were also detected by BATMAN-TCM database (Liu et al. 2016) analysis and were located in the classical AD pathway of KEGG (Figure 3). Although *PTGS2*, *BCHE*, and *ACHE* do not participate in the AD pathway, they are responsible for the regulation of memory, learning, synaptic plasticity, acetylcholine catabolic, which play an important role in AD (Table 3).

**Table 3.** The structure and targets for AD relevant Fuzi’s compounds.

Database	Chemical	Structure	Targets and AD relevant pathway
TCMSP	Arachic acid	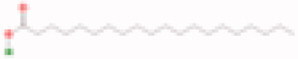	**PTGS2:** memory, learning, positive regulation of synaptic plasticity, positive regulation of synaptic transmission, glutamatergic, inflammatory response, lipoxygenase pathway, maintenance of blood-brain barrier
	14-Deoxy-11,12- didehroandrographolide	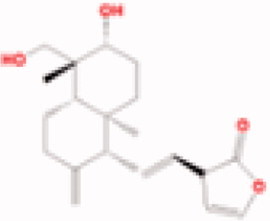	
	Delphin_qt	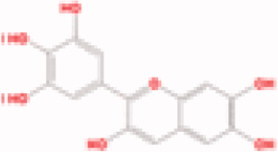	
	Deoxyandrographolide	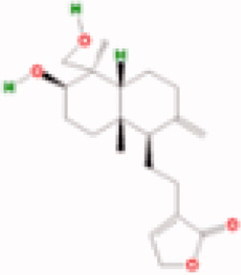	
	Myristic acid	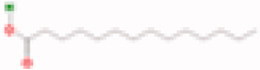	PTGS2/ **BCHE:** *acetylcholine catabolic process*, learning, negative regulation of synaptic transmission, neuroblast differentiation, synaptic transmission
	Mescaline	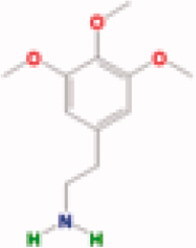	PTGS2/ **CHRM1**: adenylate cyclase-inhibiting G-protein coupled acetylcholine receptor signalling pathway, *G-protein coupled acetylcholine receptor signalling pathway*, synaptic transmission, cholinergic
	Salsolinol	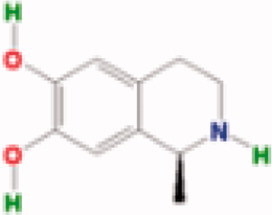	
	EIC	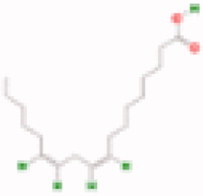	PTGS2/ CHRM1/ CHRM2
	Fuzitine	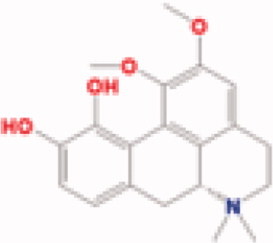	PTGS2/ CHRM1/ **ACHE:** *acetylcholine catabolic process*, acetylcholine catabolic process in synaptic cleft, negative regulation of synaptic transmission, cholinergic, synapse assembly, synaptic transmission
BATMAN-TCM	Coryneine	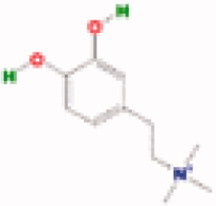	**MAPK1:***Alzheimer disease*, activation of MAPK activity, apoptotic process, axon guidance, long-term synaptic potentiation, *Activation of NMDA receptor upon glutamate binding and postsynaptic events*
	M-Aminophenol	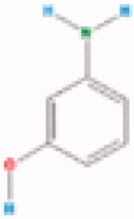	**GRIN1:***Alzheimer disease*, Glutamatergic synapse, activation of MAPKK activity, adult locomotory behavior, axon guidance, regulation of long-term neuronal synaptic plasticity, regulation of synapse assembly, synaptic transmission, glutamatergic
	Ortho-Aminophenol	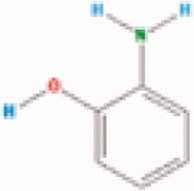	
	P-Aminophenol	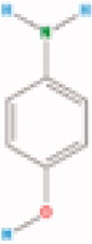	

### Compounds of Fuzi may play a key role in AD treatment

**Figure 4.**
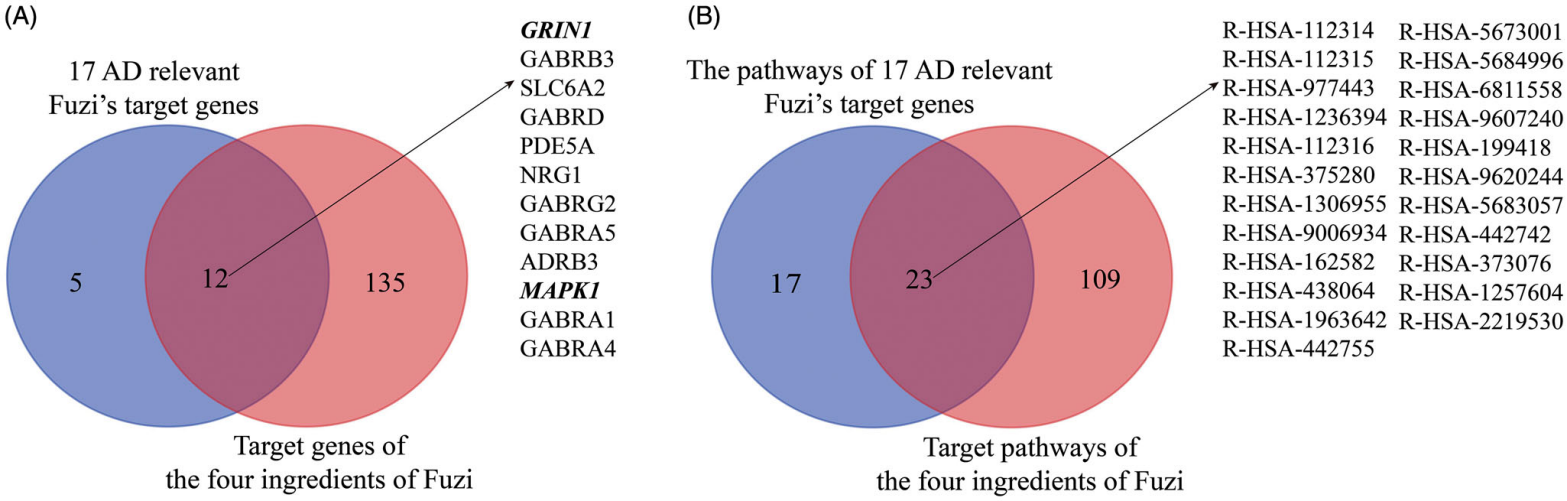
The relationship of 17 candidate genes and 4 compounds (*m*-aminophenol, *o*-aminophenol, *p*-aminophenol, and coryneine) related genes. (A) The crossover relationship of the genes. (B) The crossover relationship of enrichment pathways that the genes enriched in.

By TCMSP database (Ru et al. 2014) scanning, eight ingredients (arachic acid; 14-deoxy-11,12-didehroandrographolide; delphin_qt; deoxyandrographolide; myristic acid; mescaline; salsolinol; EIC; fuzitine) of Fuzi related to AD were figured out (Table 3). More importantly, four compounds of Fuzi ingredients targeting on *MAPK1* and *GRIN1*, which have been implicated in the regulation of neuron survival and functional pathway, were selected from the predicted list download from the BATMAN-TCM database (Liu et al. 2016). Three of them (M-aminophenol, Ortho-aminophenol, and P-aminophenol) targeting on *GRIN1*, and one (Coryneine) on *MAPK1* (related scores ≥ 20). Amazingly, except *MAPK1* and *GRIN1*, they may also target on the other AD relevant Fuzi target genes: *GABRB3*, *SLC6A2*, *GABRD*, *PDE5A*, *NRG1*, *GABRG2*, *GABRA5*, *ADRB3*, *GABRA1,* and *GABRA4* (Figure 4(A)). Enrichment analysis for all target genes (147) showed that the target genes are also responsible for axon growth, cell apoptosis, cell death, proliferation, and cell survival (Supplementary Material), and 45 pathways are shared by the four compounds and Fuzi (Figure 4(B)).

## Discussion

In this study, the AD cell models were treated with Fuzi granules at a gradient concentration (0.4–300 mg/mL) for 24 h. The cellular improvement by Fuzi showed a clear preference in different concentrations. It mainly promoted the growth of cell projections at 0.4–0.8 mg/mL, it increases the cellular proliferation from 1.6 to 100 mg/mL, and it is responsible for the toxic aspect of Fuzi in high concentration (Figure 1(A)). In further bioinformatics analysis, 17 genes were closely related to AD by combined data from BATMAN-TCM (Liu et al. 2016), Psychiatric Genomics Consortium (Lambert et al. 2013), GEO (Naoi et al. 2004), and GeneCards (Stelzer et al. 2016) databases (Figure 1(B)). Five of them were located in the important place of the classical AD pathway (Figure 3). In addition, the 17 genes all take part in pathways for the axon growth, the bi-directional regulation for cell death and proliferation, and the function of the neuronal system and five target genes of Fuzi from TCMSP database (Ru et al. 2014) were responsible for memory, learning, synaptic plasticity, acetylcholine catabolic regulating. These bioinformatics results suggest that these regulatory genes are vital for the process of Fuzi inhibit AD. Subsequently, the analysis found 8 AD relevant ingredients of Fuzi from the TCMSP database (Ru et al. 2014), and four ingredients from the BATMAN-TCM database (Liu et al. 2016), which targeted on *GRIN1*, *MAPK1* and other AD relevant genes. It suggests these ingredients may be the basis for Fuzi to antagonise AD.

### Fuzi in different concentration may be useful for the treatment of different phases of AD

In our research, Fuzi treatment with 0.4–0.8 mg/mL concentration increased the length of the cell’s projections and maintained cellular morphology. The length of SH-SY5Y cell projections was considered as the morphological marker for the SH-SY5Y cell line differentiated to own neuron function (Påhlman et al. 1981). Moreover, the bioinformatics analysis found that the target genes of Fuzi take part in the regulation of axon growth and synaptic plasticity. It implied that Fuzi might help neuron sprouting to form some new synapses to protect the function of nerve cells in the very low concentration. The current research showed that AD may originate from a cognitive decline caused by synaptic terminals loss (Ali et al. 2018), which means that the low concentration might be useful for preventing AD in the cognitive decline phase.

For the next stage of AD treatment, low to medium concentration Fuzi may be effective. Previous research pointed out that intracellular Aβ accumulation occurred before extracellular accumulation, damages not only the cell itself but also adjacent cells. With damage expanding and aggravating, large numbers of cells die, forming extracellular Aβ accumulation, which eventually leads to permanent dementia (Naoi et al. 2004). Therefore, in these stages, the neuron protection and injury inhibition are most important for AD treatment. The experiment and bioinformatics researches showed that the low to medium concentration Fuzi might target on some genes, which could regulate survival, proliferation, apoptotic, and death of cells, to improve the APP cells’ viability significantly. Particularly, Fuzi in 25 mg/mL significantly increases cell proliferation while ensuring that the cell length and cell morphology are in a good state. It means low to medium concentration Fuzi may play a positive role in these serious stages of AD. It may achieve unexpected results in the combination of existing AD drugs with Fuzi (25 mg/mL) or its active ingredients because the combination simultaneously achieves symptom relief and nerve protection.

Another notable point is that different AD relevant compounds of Fuzi have been found by TCMSP database (Ru et al. 2014) and BATMAN-TCM database (Liu et al. 2016), which plays a different role in AD regulation. Previous research on motor nerve terminal suggests that mescaline and coryneine could regulate ACh releases (Ghansah et al. 1993; Kimura et al. 1995; Nojima et al. 2000), which play a key role in AD by influencing synaptic loss and signal transform (Mangialasche et al. 2010; Ren et al. 2020; Wang et al. 2020; Zhu et al. 2020). Deoxyandrographolide activates *PI3K/AKT* pathways (Zhao et al. 2019), which are responsible for Alzheimer’s treatment by regulating the oxidative stress (Ali et al. 2018). While metabolites of salsolinol inducing apoptosis in dopamine neurons have been reported (Naoi et al. 2004). Combined the results that Fuzi boosts the cell projections in very low concentration, promotes the proliferation activity of cells in low to medium concentration, and causes injury of APP cells in high concentration (>100 mg/mL), we speculated that in different concentrations the core active ingredients of Fuzi might differ, and they regulate different pathways to anti-AD. Therefore, different Fuzi concentration may suit different treatment phases of AD.

### GRIN1 and MAPK1 may be the key target genes for Fuzi anti-AD

The current study revealed that the 17 Fuzi target genes are closely related to AD and maybe the key for Fuzi anti-AD process, especially *GRIN1* and *MAPK1*. The 17 genes are significantly differentially expressed between AD and normal samples (RMS_*p* ≤ 0.05). They posses high AD-related scores (related scores ≥ 5) in the GeneCards database (Stelzer et al. 2016). The 15 genes of them contain significant SNP sites (*p* ≤ 0.05) in AD GWAS analysis. Five genes of them directly interact with APP protein (Figure 1(B)) and take part in the regulation of axon growth, apoptosis, death and proliferation, and the function of the neuronal system (Table 2), which are all playing an important role in AD (Zhao et al. 2019). These results provide compelling evidence for the potential function of the 17 Fuzi target genes for anti-AD. Intriguingly, the analysis further showed that *GRIN1* and *MAPK1* not only directly interact with APP protein in the key point of the AD regulation pathway, but also participate in half of the AD relevant pathways that the 17 genes are involved. In addition, in the *m*-aminophenol, *o*-aminophenol, and *p*-aminophenol target to *GRIN1* showed very similar structures with carvacrol, which have therapeutic potential in preventing AD and protective effect on brain injury (Zhong et al. 2013; Ali-Shtayeh et al. 2020; Azizi et al. 2020; Shahrokhi Raeini et al. 2020).

For another, *GRIN1* and *MAPK1* have been closely related to neurodegeneration, synaptic plasticity, cell survival and AD in previous researches (Coyle et al. 2016; Preciados et al. 2016; Sun and Nan 2017; Lu and Malemud 2019). *GRIN1* protein is a critical subunit of *NMDA* (Kaniakova et al. 2012), which is the target of memantine (Robinson and Keating 2006), and plays a key role in memory and learning by regulating the plasticity of synapses (Mori et al. 2011; Wang et al. 2011). GluN1 receptors and *GRIN1* gene expression levels and location are significantly different in AD samples compared to controls (Leuba et al. 2014; Mohamed et al. 2015; Agca et al. 2020). The *MAPK1* gene is believed as one age-dependent transcriptional changing gene that involves in the abnormal hyperphosphorylation of the tau-protein, causing aggregated neurofibrillary tangles (Kálmán et al. 2009). Moreover, galantamine could treat Alzheimer’s disease by attenuating the activation of *MAPK1* (Noda et al. 2010). Taken these results together, we hypothesise that the complex regulation network with the core of *GRIN1* and *MAPK1* may play a key role in the process of Fuzi anti-AD.

Overall, our study suggests that different concentrations of Fuzi can be used in different treatment stages of AD by regulating the complex regulation network with the core of *GRIN1* and *MAPK1*. Besides, the four compounds of Fuzi targeting *GRIN1* and *MAPK1* may be the key to anti-AD medicine. However, it still requires more neurobiological and animal experiments to verify the improvement of synaptic function by low-concentration of Fuzi and the effectiveness of different concentrations of Fuzi and compounds on the body function. At the same time, the molecular mechanisms of the Fuzi and the four ingredients’ anti-AD still need more molecular, neurobiological, and animal experiments. Furthermore, since Fuzi has long-term use as a food in some areas of Yunnan province, so an epidemiological survey for these areas may help to define the relationship between Fuzi and AD.

## Data Availability

The authors confirm that the data supporting the findings of this study are available within the article [and/or] its Supplementary materials.
